# Food authentication from shotgun sequencing reads with an application on high protein powders

**DOI:** 10.1038/s41538-019-0056-6

**Published:** 2019-11-19

**Authors:** Niina Haiminen, Stefan Edlund, David Chambliss, Mark Kunitomi, Bart C. Weimer, Balasubramanian Ganesan, Robert Baker, Peter Markwell, Matthew Davis, B. Carol Huang, Nguyet Kong, Robert J. Prill, Carl H. Marlowe, André Quintanar, Sophie Pierre, Geraud Dubois, James H. Kaufman, Laxmi Parida, Kristen L. Beck

**Affiliations:** 1 Consortium for Sequencing the Food Supply Chain, San Jose, CA USA; 2 IBM T.J. Watson Research Center, Yorktown Heights, NY USA; 3 IBM Almaden Research Center, San Jose, CA USA; 4 University of California Davis, School of Veterinary Medicine, Davis, CA USA; 5 Mars Global Food Safety Center, Beijing, China; 6 Wisdom Health, A Division of Mars Petcare, Vancouver, WA USA; 7 Mars Incorporated, McLean, VA USA; 8 Bio-Rad Laboratories, Hercules, CA USA; 9 Bio-Rad, Food Science Division, Marnes-La-Coquette, France

**Keywords:** Metagenomics, Food microbiology

## Abstract

Here we propose that using shotgun sequencing to examine food leads to accurate authentication of ingredients and detection of contaminants. To demonstrate this, we developed a bioinformatic pipeline, FASER (Food Authentication from SEquencing Reads), designed to resolve the relative composition of mixtures of eukaryotic species using RNA or DNA sequencing. Our comprehensive database includes >6000 plants and animals that may be present in food. FASER accurately identified eukaryotic species with 0.4% median absolute difference between observed and expected proportions on sequence data from various sources including sausage meat, plants, and fish. FASER was applied to 31 high protein powder raw factory ingredient total RNA samples. The samples mostly contained the expected source ingredient, chicken, while three samples unexpectedly contained pork and beef. Our results demonstrate that DNA/RNA sequencing of food ingredients, combined with a robust analysis, can be used to find contaminants and authenticate food ingredients in a single assay.

## Introduction

Food ingredient authentication is important for preventing cross contamination, food fraud, and protecting food quality at each step in the supply chain. Accurate testing can improve consumer safety and protect public health. Ingredient authentication enables the manufacturer to detect variation and adulteration so that the consumer receives a product that matches written product specifications, is free of contaminants, and is safe to consume. Manufacturing equipment cross-contact and human errors are some of the reasons contributing to unintentional contamination in the food supply chain.^1^ In addition, food fraud does occur such as the 2013 discovery of undeclared horse meat in European meat products.^2^ Ingredients may be substituted for similar alternatives due to low cost or limited availability.

There is a growing effort to detect contaminants before an item enters the human food chain by leveraging molecular methods.^3–5^ DNA barcoding, PCR, and related targeted molecular methods for food authentication detect species across the plant and animal kingdoms. Many of these techniques focus on detecting specific signatures such as ribosomal RNA (rRNA), cytochrome c oxidase I (COI), or maturase K (*matK*) genes. Example use cases of these methods include food and wildlife forensic applications,^6^ authentication of plant food products,^7^ and identification of frequently mislabeled fish species^8^ and herbal supplements.^9^ Targeted gene sequencing and PCR methods may work well for testing known ingredients; however, many food products undergo a deviation from the physical form of their original food source and the final product cannot be assumed to contain only the starting material or only expected contaminants. Testing for tens or hundreds of potentially present species in a complex food matrix is not a practical approach. While PCR tests targeting multiple species are available, for example in direct-multiplex PCR for simultaneous pork, lamb, chicken, ostrich meat, horse meat and beef testing,^10^ primer design and unexpected cross-reactivity with other potential matrix species are some of the challenges associated with such tests.^11^ A recent review on molecular methods for food authentication^3^ discusses the associated challenges in sample preparation, targeted amplification and analysis for use in food testing. Speranskaya et al. noted the challenges with existing approaches for food authentication and discussed prospects of using high-throughput sequencing for testing the composition of food products.^12^

High-throughput nucleic acid sequencing, combined with robust bioinformatic analysis, has the potential to replace or augment current tests for verifying food ingredient composition by detecting contaminants without prior assumptions of the expected content. This includes detection of eukaryotic species present in trace amounts (e.g., at concentrations <1% of the total composition). Other efforts in the space of food authentication by shotgun metagenomics include the All-Food-Seq pipeline^4^ and recent research on identifying species in herbal mixtures.^13^ In addition to the food matrix composition, metagenomic sequencing importantly yields a snapshot of the microbial content and possible pathogens.

We hypothesized that metagenomics will overcome the limitations of other molecular methods to provide an accurate method to simultaneously detect multiple contaminants. Use of metagenomic sequencing to authenticate complex food types is a new approach that requires bioinformatic best practices to be developed. To advance this approach, public metagenome sequences and custom simulated in silico food sequence datasets were examined to develop and calibrate a food authentication pipeline that produces accurate eukaryotic species identification and relative quantification from high-throughput metagenomic sequencing reads, across plant and animal sources. This approach has many applications that include adulteration and hazard detection, quality control, e.g., when working with new suppliers, and detection of anomalous samples which could indicate an issue in the food supply chain.

**Fig. 1:**
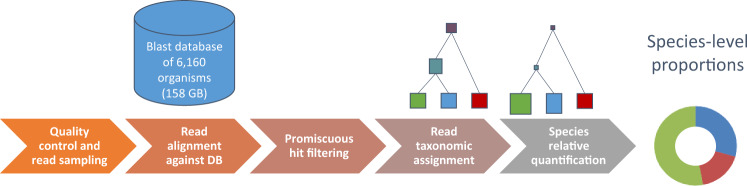
Pipeline applied to food sample sequencing data to determine matrix species and their relative proportions. In the taxonomic assignment step with exemplary diagram, reads are placed on the lowest common ancestor (LCA) of the nodes that they hit, in case of multiple hits per read. In the relative quantification step the read counts at internal nodes are re-assigned to the species at the leaf nodes

In this paper, we describe a food matrix authentication bioinformatic pipeline, Food Authentication from SEquencing Reads (FASER), for use with high-throughput total DNA or RNA sequencing and demonstrate its applicability for use in food (Fig. 1). One key component of the pipeline is a comprehensive stand-alone BLAST^14^ search index built on a whole genome reference collection containing 6,160 unique plant and vertebrate organisms (158 GB total size), which enables accurate species assignment directly from sequencing data. Together the database and bioinformatic analysis steps allow relative quantification of single and multi-ingredient samples across diverse plant and animal species. We applied FASER to 11 experimental and 5 in silico datasets with expected compositions and demonstrated accuracy achieving a 0.4% median absolute difference between observed and expected relative proportions of the true positive species. On average, the observed combined relative proportion of true positive species per sample was over 99%. We demonstrated that utilizing as few as 150,000 paired end (100–150 bp length) sequencing reads was sufficient to achieve this accuracy on both in silico simulated sequence mixtures and publicly available experimental data. To further improve detection of low abundance components, the number of reads used in the analysis was increased to ~500,000 for authenticating food samples from raw sausage meat and protein powders. To examine the use of this approach in the food supply chain, we applied FASER to a collection of 31 raw factory ingredient high protein powder samples using total RNA sequencing. We observed the eukaryotic food ingredient to be poultry in most cases as expected, with the unexpected observation of pork and beef in three samples. Collectively, this work provides a sensitive and accurate untargeted method to detect contamination directly from total DNA or RNA sequencing.

## Results

To test the accuracy of the pipeline across a diverse set of ingredient types and sequencing methods, validation was done using 5 simulated and 11 experimentally sequenced datasets of expected composition. Among these sequenced datasets were four preliminary factory ingredient samples: two biological samples for which both RNA and DNA sequencing was completed. The pipeline was additionally applied to 31 commercial raw material samples of a high protein powder factory ingredient to test the authentication of food ingredients and detection of contaminants.

### Determining the number of reads required for species detection

Choosing the number of reads to be sequenced for a specimen occurs upstream of the FASER pipeline and is typically limited by sequencing cost. Our underlying assumption is that due to laboratory costs associated with sequencing, the number of sequenced reads is desired to be as low as possible, while still enabling detection of species that are present above a desired threshold, e.g., above 1% of the sample total. The time and cost of bioinformatics processing is low (in the order of hours for data analyzed in this paper) compared with sequencing more reads of a sample, or sequencing replicates of the same sample. Details on the compute environment and timing of the FASER pipeline are included in Supplementary Methods.

In order to identify a breadth of eukaryotic species without prior knowledge of the expected ingredient(s), a large reference database containing the appropriate representative sequences must be used; however, querying millions of sequencing reads (as is common in a single sequencing run output) against such a database will be very slow and may not be necessary for food matrix authentication. In the All-Food-Seq pipeline, Ripp et al.^4^ used subsets of 500,000 reads (from 16 million sequenced reads) for food matrix authentication. We ultimately chose to use a similar number of reads, but also demonstrated using a mathematic model how subsampling reads for increased speed of matrix authentication affects the species detection capability and accuracy.

**Fig. 2:**
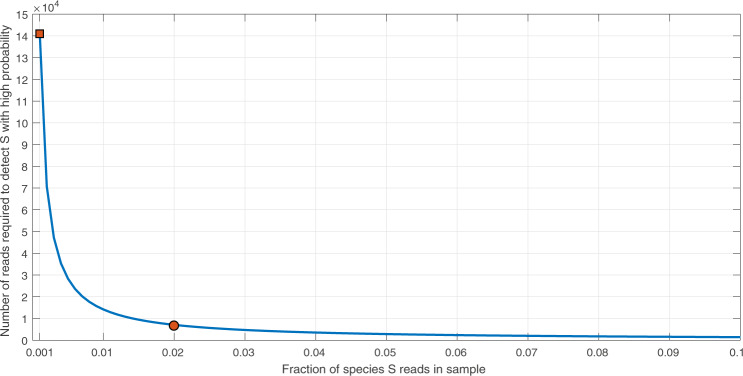
Illustration of the minimum size of a subsample to obtain a desired limit of detection. The required number of reads is shown as a function of frequency of species *S* (in the full sample). In this example with a total number of *N* = 300 million reads, we desire with high probability *P* (here *P* ≥ 0.9999) to have limit of detection at least *L* = 100 sampled reads coming from species *S* when *S* is present. For example, when frequency of *S* is 0.1% (*x* = 0.001), a subsample of 141,499 reads from the total 300 million reads is required (marked with a square). When frequency is *S* is 2% (*x* = 0.02), fewer than 10,000 reads are required (marked with a circle)

**Fig. 3:**
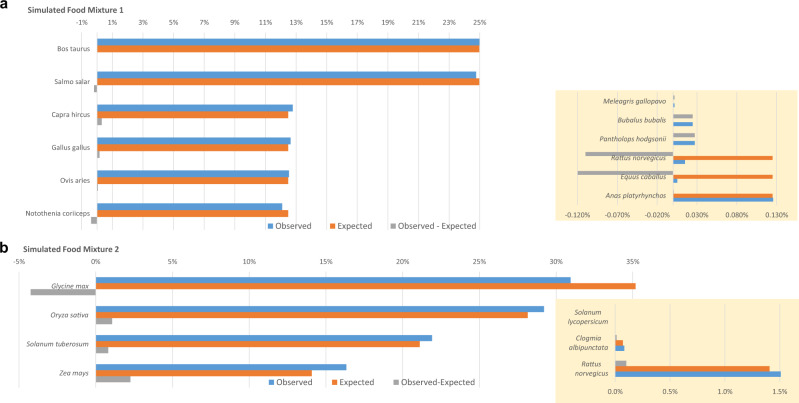
FASER pipeline accuracy on two simulated food mixtures. **a** Simulated food matrix 1. **b** Simulated food matrix 2. Insets are shown separately to accommodate different scales. Details regarding the input genomes are given in Supplementary Table 6

The modeled read subsampling and probability of species detection capability are illustrated in Fig. 2. For a dataset with 300 million reads, such as the high protein powder dataset analyzed in this paper, 450,000 reads represent 0.15% of the total sequencing information available from a sample. Based on Eq. (1), this subsample size supports detection of at least 100 sequencing reads from any species with frequency as low as 0.032% in the full sample with high probability (*P* ≥ 0.9999), thus allowing for sensitive detection of constituent species. Supported by this calculation for species detection, we analyzed 0.15% of the sequencing reads for the high protein powder samples, resulting in a similar number of reads as Ripp et al.^4^ We found that fewer reads were sufficient to test both the species detection hypothesis as well as to quantify the species proportions in the in silico datasets and in single ingredient public datasets. Thus, as few as 150,000 reads for the simulated datasets (Fig. 3, Tables 1 and 2) and the single ingredient datasets (Table 3) was used to test the detection and accuracy limits of the pipeline on low pass sequencing data.

**Table 1:** Food matrix authentication results from 150,000 simulated reads of single species food matrix samples from (A) chicken (*Gallus gallus*), (B) pork (*Sus scrofa*), (C) beef (*Bos taurus*)

Taxon name	Common name	TaxId	FASER: 10^−40^ hits	10^−10^ hits	FASER: % Assignment with 10^−40^	% Assignment with 10^−10^
*A: Species assignment of simulated chicken food matrix*						
*Gallus gallus*	Chicken	9031	116,085	119,994	99.98%	99.98%
*Meleagris gallopavo*	Turkey	9103	11	18	0.01%	0.01%
*Coturnix japonica*	Japanese quail	93934	7	9	0.01%	0.01%
*Anser cygnoides*	Swan goose	8845	0	1	0.00%	0.00%
Total			116,103	120,022		
*B: Species assignment of simulated pork food matrix*						
*Sus scrofa*	Pork	9823	107,075	115,178	100.00%	100.00%
*Orcinus orca*	Killer whale	9733	0	1	0.00%	0.00%
Total			107,075	115,179		
*C: Species assignment of simulated beef food matrix*						
*Bos taurus*	Beef	9913	114,699	117,906	99.91%	99.88%
*Bubalus bubalis*	Water buffalo	89462	85	121	0.07%	0.10%
*P*. *hodgsonii*	Tibetan antelope	59538	10	13	0.01%	0.01%
*Capra hircus*	Goat	9925	5	9	0.00%	0.01%
*Ovis aries*	Sheep	9940	0	3	0.00%	0.00%
Total			114,799	118,052		

Paired-end reads were simulated from the respective genomes listed in Supplemental File 3 (highlighted in blue). Blast e-value thresholds 10^−40^ and 10^−10^ were applied; 10^−40^ is used in the FASER pipeline. The number of read hits are shown as well as the percentage of simulated reads that were assigned to the listed species

**Table 2:** Novel BLAST promiscuous hit filtering on 1000 paired-end simulated *Bos taurus* reads

		With promiscuity filter	Without filter
Species name	Common name	Observed%	Observed%
*Bos taurus*	Beef	100.00%	92.55%
*Bubalus bubalis*	Water buffalo	–	2.21%
*Bos mutus*	Wild yak	–	1.86%
*Bos indicus*	Zebu	–	1.51%
*Bison bison*	Bison	–	1.28%
*Ovis aries*	Sheep	–	0.23%
*Pantholops hodgsonii*	Chiru	–	0.23%
*Capra hircus*	Goat	–	0.12%
Number of reads with hits		784	859

Left: after filtering, right: before filtering

**Table 3:** FASER results on experimental samples

Sample name	Sequence type	Expected species	% Observed expected species	Difference of observed from expected	Other species observed at >0.1% (common names shown where available)	Sample identifier
Chicken embryo	RNA (polyA selected)	*Gallus gallus*	98.34%	−1.66%	Turkey 1.02%; Japanese quail 0.43%; Green junglefowl 0.11%	SRR1804235
Pork ovaries	RNA (polyA selected)	*Sus scrofa*	96.38%	−3.62%	Water buffalo 0.47%; Minke whale 0.34%; Sperm whale 0.26%; Orca 0.20%; Alpaca 0.16%; Baiji doplhin 0.16%; Walrus 0.15%; Wolf 0.15%; Chiru 0.14%; Bottlenosed dolphin 0.11%	SRR6236882
Yellowfin tuna muscle	RNA (polyA selected)	Thunnus albacares*	99.80%	−0.20%	*Bluefin tuna reported Black rockcod 0.13%	SRR4436659
Carp spleen	RNA (polyA selected)	*Cyprinus carpio*	96.47%	−3.53%	*Sinocyclocheilus graham* 1.11%; *S. anshuiensis* 1.10%; *S. rhinocerous* 1.05%; *Acrossocheilus monticola* 0.18%	SRR3239506
Rice root	RNA (polyA selected)	*Oryza sativa*	99.57%	−0.43%	Date palm 0.14%; Oryza brachyantha 0.11%; Human 0.11%	SRR7079262
Maize leaf	RNA (polyA selected)	*Zea mays*	99.97%	−0.03%	–	ERR712359
Poultry meal (paired samples)	Total RNA	*Gallus gallus*	99.96%	−0.04%	–	MFMB-03
	Total DNA		99.71%	−0.29%	Maize 0.17%	MFMB-08
Meat and bone meal (paired samples)	Total RNA	*Bos taurus*	99.72%	−0.28%	Chicken 0.28%	MFMB-02
	Total DNA		99.51%	−0.49%	Chicken 0.43%	MFMB-06

Results on six single ingredient polyA-selected RNA datasets from NCBI and on four high protein powder paired total DNA and RNA samples (MFMB-02 through MFMB-08). Additional dataset details are given in Supplementary Table 1

### Parameter calibration and optimization

We examined the BLAST-matching parameter settings to test the hypothesis that metagenome sequencing data can be used to identify matrix members via a BLAST search. Similar sensitivity was observed with e-value parameters of 10^−10^ and 10^−40^ (Table 1). The 10^−40^ threshold yielded fewer false positive hits in simulated testing as compared with 10^−10^ while improving computational efficiency due to a smaller output size without compromising the results. Therefore, this BLAST parameter was chosen for further use in this study.

We considered that if the vast majority of the alignments assigned to species *S* are equally good to one or more other species, then the reads aligning with *S* may actually come from other species causing a false positive result. Accordingly, we developed a downstream *promiscuity filtering* process to further reduce the number of false positive species. The promiscuity filtering of taxa improved accuracy for *Bos taurus* (cattle) (Table 2) in the case where phylogenetically similar species may occur in the database, such as *Bos mutus* (wild yak), *Bos indicus* (zebu), *Bubalus bubalis* (water buffalo), and *Bison bison* (bison). With promiscuity filtering, only alignments to *B*. *taurus* were reported, consequently increasing the relative frequency of *B*. *taurus* in the results from 92.55 to 100% (matching the expected composition). In addition, we found that the required minimum threshold of 10% unique alignments specifically allowed unique identification of each species and allowed more reads to be unambiguously assigned to a species.

### Accuracy evaluation with in silico simulated data

Three in silico constructed datasets were evaluated using known compositions of (i) single food sources, (ii) only microbes, and (iii) complex microbiomes with plants, animals, and microbes to examine the accuracy of matrix authentication from sequencing data. These datasets were all processed with FASER without a priori assumptions of the samples’ content.

In the first simulated experiment, datasets were generated to model common food ingredient species related to the collection of 31 high protein powder samples (see the section ‘High protein powder factory ingredient sample collection and sequencing’). The product specifications indicated that the ingredient’s primary animal source was chicken (*Gallus gallus*). However, other livestock species could be unintentionally present due to challenges in the supply chain. To mimic this situation, we simulated and analyzed reads from three common livestock species—chicken (*G*. *gallus*), beef (*B*. *taurus*), and pork (*Sus scrofa*) (Table 1). Overall, fidelity between the reference genome and simulated reads was achieved. However, a small fraction (<0.1%) of the simulated reads were observed to better match other genomes than the originating one. This small amount of false positive hits suggested that low levels of false positive hits matching turkey (*Meleagris gallopavo*) and quail (*Coturnix japonica*) may be expected in results on chicken samples (as well as false positive water buffalo and sheep hits on beef samples). This simulated experiment’s results demonstrated FASER achieved >99.9% detection capability of the expected species content in each case.

In the second simulated experiment, we tested the assumption that microbial reads do not interfere with matrix species identification, as they are expected to be a minor proportion of the total reads compared with matrix reads in food samples. We examined the rate with which microbial reads were falsely assigned to eukaryotic plant and animal genomes. For this we used simulated reads from a microbial reference genome database (NCBI RefSeq Complete,^15^ ~7800 microbial genomes) and processed them with FASER. From 150,000 simulated microbial reads, 204 (0.14%) were assigned to a matrix species. Chiru (*Pantholops hodgsonii*) was identified by 0.04% of the total input reads and was the largest proportion of false positive matrix content reported from the microbial content. No other matrix species had more than 50 BLAST alignments assigned. We further confirmed that with 300 and 450 K simulated microbial reads the observed matrix species hits had the same low rate (0.14%). Based on these results, when microbial reads comprised a minor proportion of the total sequencing reads (e.g., <1%), the expected false positives from microbes was <0.0014%.

In the third simulated experiment, FASER was evaluated on two in silico food microbiomes where the matrix content was constructed from multiple sources e.g., chicken (*G.* *gallus*), pork (*S*. *scrofa*), and soy (*Glycine max*) with the microbiome representing 0.11–0.19% of total sequences. The tests were designed to model realistic food microbiomes and evaluation of the data demonstrated the accuracy of FASER (Fig. 3, details of the simulation are given in the section ‘Constructing simulated datasets’). The median absolute difference was 0.84% between the expected and observed proportions for each input species. In order to compare the expected and observed compositions, we used the *χ*^2^ test. The test showed no significant difference (*P* ≥ 0.99; data not shown) between the expected and observed species compositions indicating that FASER indeed accurately identified the expected contents.

### Accuracy evaluation on single species sequencing data

The accuracy of species assignment was tested on real mRNA sequencing data from single species experiments retrieved from NCBI Sequence Read Archive (SRA)^16^ (chicken (*G*. *gallus*) embryo, pork (*S*. *scrofa*) ovary, tuna (*Thunnus albacares*) muscle, carp (*Cyprinus carpio*) spleen, rice (*Oryza sativa*) root, and maize (*Zea mays*) leaf tissues; see Supplementary Table 1 for dataset information). For all datasets, >96% of the species-matching reads identified the expected matrix species (Table 3) and there were no false positive species representing >0.5% of the reads except in carp spleen and chicken embryo.

A small fraction of chicken embryonic mRNA reads matched to turkey (1.02%) and quail (0.43%), as was also detected in the simulated data experiments (Table 1). Note that the chicken sequences were directly sampled from fertilized eggs and had no turkey or quail RNA present. This demonstrated that the real data results closely matched the in silico observations, thus validating the simulation framework for modeling real sequencing data.

In the carp spleen sample, 3.26% of the reads were assigned among three other fish species in the genus *Sinocyclocheilus* that belongs to the same family as carp, *Cyprinidae*. In addition, in the tuna sample, the expected species was *T*. *albacares* (yellowfin tuna), but FASER reported 99.8% *T*. *orientalis* (bluefin tuna). The analysis was correct in assigning tuna but was unable to differentiate yellowfin from bluefin as only bluefin tuna was present in the BLAST database. The carp and tuna results highlight the need for a diverse reference database that includes the relevant species of interest to achieve the most accurate identification. Overall the results in Table 3 demonstrated high accuracy, 0.36% median absolute difference of observed from expected species content, and agreed with the observations from simulated data. The difference between all expected vs. observed values was not significant (*χ*^2^ test *P* ≥ 0.99; data not shown), indicating FASER accurately identified the expected contents.

### Accuracy evaluation on a complex food metagenome of known composition

The closest pipeline for food metagenomes analysis found in the literature is All-Food-Seq.^4^ This pipeline was published with an application on DNA metagenome reads from a raw sausage mixture, matched against a small reference database (19 genomes, Table S1 in Ripp et al.^4^). The sausage contained a known composition, by weight in grams: 55% sheep, 35% beef, 9% pork, and 1% horse meat. In addition to the raw meats, the sausage mixture also contained material from 11 plant species at trace amounts to test the pipeline’s ability to detect low abundance contaminants. The highest divergence from the target proportion observed by All-Food-Seq was for pork (−1.79%).

We analyzed all the available 409,616 paired reads (MiSeq dataset SRR1745838 from NCBI) to compare FASER with All-Food-Seq on the same input data. FASER correctly identified the main ingredients and their relative proportions: sheep, beef, and pork, and additionally found 1% horse meat, with a median absolute difference of 0.44% (Table 4). The highest divergence from the target proportion in our analysis was also pork (−2.25%), followed by beef (+0.44%). Of unexpected matrix components, we observed goat reads at 1.83%. Goat was not included in the database used for All-Food-Seq; therefore, not present in their results and a false positive in this analysis. The simulated testing supported a small fraction of cattle (Table 1) and sheep reads (Supplementary Table 2) matching goat better than the originating genomes.

**Table 4:** FASER results on experimental food mixture

Species name	Common name	BLAST hits count	Observed	Expected	Observed − expected
*Ovis aries*	Sheep	149,726	54.07%	54.49%	−0.42%
*Bos taurus*	Beef	97,224	35.11%	34.67%	0.44%
*Sus scrofa*	Pork	18,459	6.67%	8.92%	−2.25%
*Equus caballus*	Horse	3048	1.10%	0.99%	0.11%
*Capra hircus*	Goat	5068	1.83%	0%	1.83%
*Bubalus bubalis*	Water buffalo	1930	0.70%	0%	0.70%
*Pantholops hodgsonii*	Tibetan antelope	1132	0.41%	0%	0.41%
Total ALL species (incl. those not listed here)		276,912	99.88%	99.068%	

Accuracy evaluation of FASER on DNA data from All-Food-Seq raw sausage meat mixture experiment. Percentages for expected (based on ingredient weights) vs. observed (based on fraction of species-level BLAST hits) are shown. Species with at least 100 hits are included in the table. The remaining 0.932% expected content is from plants (see Supplementary Table 3)

Since the All-Food-Seq publication reported results on plant contaminants only at family level, we compared them with FASER results after summing up the counts at family level (Supplementary Table 3). In addition, we present the plant content at species level directly. FASER detected 6/8 of the low abundance plant contaminants at family level. For the remaining two families, the All-Food-Seq pipeline detected only 1 and 3 reads, out of the total 409,616 paired reads. FASER detected more walnut and fewer mustard reads while other family-level counts were comparable to All-Food-Seq. FASER detected 6/11 of the low abundance plants at species level, providing increased taxonomic resolution in contaminant detection compared with All-Food-Seq results at family level. For example, in the family *Fabaceae* (legumes), FASER accurately detected 10-fold more lupine than soybean species reads.

### Summary of accuracy evaluation

In summary of the above described validation work, we applied FASER to eleven experimental (Tables 3 and 4) and five in silico validation datasets (Fig. 3 and Table 1) representing single and mixed ingredient foods with expected compositions and demonstrated accuracy achieving a 0.4% median absolute difference between observed and expected relative proportions of the true positive species (mean 1.0%, std. 1.4%, min. 0.0%, max. 5.9%). On average, the combined relative content of true positive species proportions per sample was over 99% (mean 99.1%, std. 1.3%, min. 96.4%, max. 100.0%), and the median number of false positive species observed was 1 (when considering species with relative proportions >0.1%). Comparing all expected vs. observed values by the *χ*^2^ statistic, no significant differences (*P* ≥ 0.99) were observed across all detected species.

### Authentication of ingredients in high protein powder sequencing data

With the robust results from multiple in silico and public dataset analyses, we progressed to analyze high protein powder samples obtained from the food supply chain to examine ingredients that were labeled to be from a single animal source.

FASER was first applied to four preliminary samples: paired DNA and total RNA sequenced from the same biological samples, to test the pipeline consistency across different types of sequencing. The samples were collected from raw factory ingredients that were stated to contain chicken and beef high protein powders (Supplementary Table 4): MBMB-03 RNA and MFMB-08 DNA for poultry meal, MFMB-02 RNA and MFMB-06 DNA for meat and bone meal. Both the DNA and RNA samples were found to contain >99% of the expected ingredient for chicken meal and meat and bone meal, with RNA yielding 0.2% fewer false positive hits than DNA (Table 3).

**Fig. 4:**
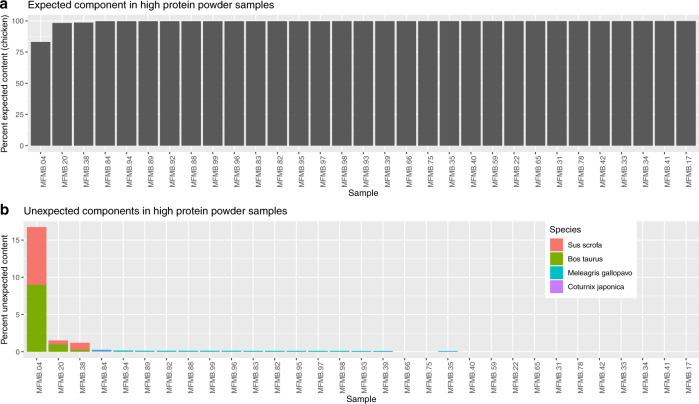
Raw high protein powder (poultry meal) samples’ FASER results showing unexpected non-chicken components. **a** Percentage of expected content (chicken). **b** Percentage of unexpected content showing species with relative proportion >0.1% of total matrix composition. Content from *Bos taurus* (beef) and *Sus scrofa* (pork) is detected for samples MFMB-04, MFMB-20, and MFMB-38

A set of 31 high protein powder samples (MFMB-04 and MFMB-17 through MFMB-99) derived from poultry were subsequently used for deep total RNA sequencing with >300 million reads per sample. These sequences were then examined to determine the ingredient composition and detect possible contamination in a single analysis to replicate an industrial use case. Using the developed sequencing approach and analysis pipeline we observed that in 28 of 31 samples 99.7–99.9% of the species-assigned sequence alignments matched chicken, the stated ingredient source (Fig. 4 and Supplementary Data). Sequence alignments matching other avian species were detected (0.08–0.26% assigned to turkey and quail) in all samples, which was expected based on the results from in silico simulations (Table 1) and chicken embryo sequencing data (Table 3), suggesting that conserved sequence content may account for the assignments to the other avian species.

In three high protein powder samples—MFMB-04, MFMB-20, and MFMB-38—pork and beef were detected in addition to the labeled chicken ingredient. The highest proportions were detected in sample MFMB-04, 7.7% pork (*Sus scrofa*) and 9.0% beef (*Bos taurus*). We further confirmed the presence of chicken as well as the presence of the observed contaminants beef and pork in an additional analysis using Bowtie 2 to align the RNA sequencing reads to chicken, bovine, and pig genomes. For this analysis we examined reads from three chicken-only samples and the three contaminated samples (Table 5). For MFMB-04, we found that 6.1% of the alignments were exclusive to the pig genome, 8.8% were exclusive to the bovine genome, and 79.2% were exclusive to the chicken genome (the remaining 5.9% of sequences aligned to more than one genome). The distribution of these reads confirmed contamination from beef and pork, supporting the matrix relative quantification obtained with FASER (Fig. 4). By leveraging an additional targeted analysis with highly specific alignment settings, three eukaryotic genomes were confirmed to be present in the samples. Thus, the authentication pipeline reported the expected ingredient and unexpected contaminants in real factory ingredients.

**Table 5:** High protein powder sequences mapping to observed source genomes

	MFMB-04	MFMB-20	MFMB-38	MFMB-39	MFMB-83	MFMB-95
TOTAL concordant hits	952,168	965,429	967,969	960,505	977,856	974,453
TOTAL exclusive hits %	94.10%	95.74%	96.23%	95.32%	92.72%	93.30%
Chicken exclusive hits %	79.19%	94.36%	95.24%	95.29%	92.67%	93.26%
Pork exclusive hits %	**6.09%**	**0.42%**	**0.71%**	0.02%	0.03%	0.02%
Beef exclusive hits %	**8.82%**	**0.96%**	**0.29%**	0.01%	0.03%	0.02%

Confirmation of poultry meal contamination by read mapping to genomes from each observed food matrix source (chicken, pork, beef) from three matrix-contaminated (MFMB-04, MFMB-20, MFMB-38) and three chicken-only (MFMB-39, MFMB-83, MFMB-95) high protein powder (poultry meal) samples. Exclusive hits mapped to only one of the three genomes. Numbers in bold indicate increased contaminant mapping rates compared with chicken-only samples

## Discussion

Bridging the gap between current targeted tests, such as PCR or ELISA,^17^ and high-throughput sequencing for use in regular testing processes and standards is an emerging challenge for the food industry. Current molecular methods for detecting food contamination include restriction fragment length polymorphism (PCR-RFLP), random amplified PCR, multiplex-PCR, DNA hybridization, and DNA barcoding.^3^ Each of these methods have shortcomings that include quantification accuracy, amplification bias, the necessity of prior knowledge of a contaminant for use with a pre-defined target sequence for primer development, and experimental inactivity due to common food additives and secondary metabolites.^3^ Analytical fingerprinting techniques such as mass spectrometry^18^ and chromatography^19^ are also being explored for food authentication, with their associated challenges including the expense of building and curating databases of reference materials.^20^

As the food industry continues to evolve and evaluates more advanced molecular methods, shotgun metagenome and whole genome sequencing of food and pathogens are becoming more widely adopted. These approaches provide deeper information than targeted tests in a single analysis and are becoming accepted for use in food safety settings with early caution as the implications of finding unexpected observations may be false positive estimations based on limited sequence information.^4,5,21^ In this study, we used data from total RNA sequencing of factory ingredients to examine whether multiple food contaminants can be detected in a single analysis with high accuracy.

In order to bring sequencing technology into standard analytical methods for food, it needs to provide robust and actionable information that is anchored in accuracy and detection limits that are reasonable for the industry. Accuracy of data analytics is of the utmost importance because use of inaccurate results could lead to incorrect conclusions and harm consumer safety or initiate unnecessary regulatory action. Bioinformatic accuracy of food matrix authentication and microbial profiling continue to hinge on the completeness of existing reference databases and proper calibration of existing and new tools against this type of data. Comprehensive use of improved public reference data and careful bioinformatics for interpreting the sequence data are both required for accurate validation of food matrix composition.

In highly processed commercial food products, observed matrix species’ DNA content linearly correlates with the mass of that species.^22^ This shows promise for translating the resulting sequencing read proportions into ingredient weight proportions in a food sample. Normalization by genome size or using an experimentally determined normalizing factor per species may be required to accurately quantify ingredient proportions in complex food samples. Encouragingly, relative DNA contents per ingredient directly match their content by weight in the example of the raw sausage meat calibration data provided by All-Food-Seq publication.^4^

Here, we define a matrix authentication pipeline, FASER, that is motivated by applications in food safety yet is relevant and useful for any intact eukaryotic matrix signal where the sample composition may be unknown or requires confirmation. With such pipelines, automated testing for food composition as well as detection of contaminants and adulterants becomes feasible. The ability to detect expected food ingredients and unexpected content depends on a comprehensive database. The reference used in this paper contained over 6,000 plant and animal sequences. The pipeline achieved relative quantification of single and multi-ingredient samples with 0.4% median absolute difference between observed and expected species relative proportions when tested with 16 simulated and experimental datasets. In each dataset, the sum of the true positive species’ proportion was >96% (Tables 1, 3 and 4, and Fig. 3). The expected vs. observed compositions were also similar according to the *χ*^2^ statistic. The observed low abundance false positives, such as turkey and quail in chicken data and goat in the sausage mixture, could be explained by conserved content between the sequenced genome and others in the database, deviation of the sequenced genotype from the reference genome, as well as sequencing errors. False positive species identification could be further reduced by post-processing the sequence reads assigned to minority components. One approach would be to contrast the observed relative abundance against expected relative abundance from simulated and experimental benchmarks derived from the most abundant species (such as those presented in Tables 1 and 3).

In this proof-of-concept study we developed a pipeline for food authentication from shotgun sequencing data, tested it on various tissue types from experimental public studies, and applied it on raw high protein powders. While FASER could be applied on any food, agricultural, supply chain or environmental sample, appropriate benchmarking and validation of the sequencing approach with the respective sample type should first be considered. Additional calibration studies of high-throughput sequencing of food, such as the sausage experiment,^4^ would yield valuable insights into the overall applicability of this approach. Authenticating other types of food sources beyond those tested here could involve challenges in nucleic acid extraction and sequencing, the availability of relevant reference genomes, and in separating signals from food matrix species with similar sequence content.

When analyzing real food data with the FASER pipeline, the discovery of unexpected content in a collection of 31 raw high protein powder samples highlights the detection power of sequencing food samples (Fig. 4). These observations also highlight the real risk of inadvertent cross contamination in the food supply. This information would not be readily detected using traditional testing methods such as macronutrient profiling or targeted molecular tests. In ongoing work we are examining the microbial content of the 31 samples whose eukaryotic composition was analyzed here. Achieving species-level accuracy is critical for food safety and quality as it will enable detection of contaminants even if present in trace quantities. We demonstrated these abilities with public raw sausage meat data^4^ with a known composition, detecting 6 out of 11 of added very low abundance plant species (some with expected quantities of 0.003% of total sample) (Supplementary Table 3).

We showed how food matrix components from mixed plant and animal origins can be identified and quantified using short sequencing reads in the few hundreds of thousands range. This is in agreement with suggested practices by the All-Food-Seq publication where <500,000 paired sequencing reads were used to identify species in the sausage data.^4^ Compared with All-Food-Seq, FASER employed a more comprehensive database (19 vs. 6,160 eukaryotes) and reported results at species level in a single analysis across the entire species collection without prior knowledge of the expected ingredients or contaminants (Table 4). All-Food-Seq only reported family-level results for the low abundance plants included in the sausage calibration experiment, while FASER reported species-level proportions. In addition, FASER automatically handled reads aligning to multiple taxa using a known taxonomy, unlike All-Food-Seq that required manually defining related sequence groups on a case-by-case basis.

Taken together, these results support the utility of FASER as a robust pipeline for eukaryotic species identification that enables simultaneous detection of multiple contaminants and authentication of ingredients from food nucleotide sequencing. This work provides a process to accurately evaluate nucleic acid sequencing data for confirmation and identification of matrix components. This serves as a key step towards bridging the gap between targeted tests that require prior knowledge of the contaminant and the successful integration of shotgun sequencing in standardized food safety testing procedures where any contamination can be identified. A risk management model is a natural usage and next step for this work.

## Methods

### High protein powder factory ingredient sample collection and sequencing

Two sets of preliminary samples of paired DNA and total RNA of high protein powder factory ingredients were sampled and sequenced from poultry meal and meat and bone meal. Subsequently, high protein powder factory ingredient samples were collected and total RNA sequenced from 31 raw poultry meal samples. The total sequence collection thus consists of 35 samples (Supplementary Table 4).

High protein powder (HPP, 2.5 kg) samples were collected from a train car in Reno, NV between April 2015 and February 2016 in four batches and shipped to the Weimer lab at the University of California, Davis (Davis, CA). Each HPP sample was composed of five subsamples from random locations within the train car prior to shipment. On the day of arrival 0.2 g of powder was added to 2 mL of Trizol LS (Ambion by Life Technology, Carlsbad, CA). After complete mixing, the samples were used to extract total RNA as described by Chen et al.^23^ and total DNA as described elsewhere.^24–29^ Total RNA purity (A_260/230_ and A_260/280_ ratios ≥ 1.8) and integrity were confirmed with Nanodrop (Nanodrop Technologies, Wilmington, DE, USA) and BioAnalyzer RNA Kit (Agilent Technologies Inc., Santa Clara, CA, USA).^23^ Subsequently, cDNA was constructed using RNA (4–15 µg total input) and SuperScript Double Stranded cDNA Synthesis kit (Invitrogen, Catalog no. 11917-020, Life Technology, Carlsbad, CA).

Sequencing libraries using HyperPrep Plus (Kapa BioSystems, Wilmington, MA, USA) cDNA were constructed as described previously^23,30,31^ with an insert size between 300 and 400 bp. Library quantification was done using qPCR (Library Quantification kit catalog #KK4824, Illumina, San Diego, CA) prior to submission for sequencing at BGI@UC Davis (Sacramento, CA). The Illumina HiSeq 4000 (San Diego, CA) was used with 150 paired-end chemistry for each sample except the following: HiSeq 2000 with 100 paired-end chemistry was used for the four preliminary samples, and HiSeq 3000 with 150 paired-end chemistry was used for MFMB-04 and MFMB-17. All of the sequences generated in this study are available via the 100 K Pathogen Genome Project BioProject (PRJNA186441) (see Supplementary Table 4 for a complete list of accession numbers for each sample).

### Constructing a comprehensive database of plants and animals

Sequences from NCBI’s RefSeq 81 genomic collection^15^ representing vertebrates and plants were downloaded on March 13, 2017. Organisms labeled “vertebrate_mammalian”, “vertebrate_other”, and “plants” within the NCBI RefSeq collection were selected, at that time comprising 11,623,393 sequences from 6160 unique organisms (taxonomic identifiers).^15^ In addition, we identified as missing from RefSeq and subsequently added genome assemblies of three major food organisms (tuna, barley, wheat) and two potential food contaminants (cockroach, drain fly) to the plant and animal database (from NCBI or Ensemble databases; details in Supplementary Table 5). Per the standard approach recommended in the BLAST manual^32^, we replaced (hard masked) low complexity regions in the reference database with a non-ACGT character using dustmasker^33^ (v1.0.0 with default parameters). This curated collection of sequences (matrix authentication database) was then used to create a BLAST (v2.6.0) nucleotide database for food ingredient authentication and contamination detection.

### Determining the number of reads required for accurate species identification

To identify trace components of a food matrix, deep sequencing is required. To improve system performance, a random subsample of reads can accelerate detection of dominant components. We used random subsamples both to accelerate ingredient authentication and contaminant detection, and to establish quantitatively the number of reads required for accurate identification of minority components. To quantify the minimum number of reads necessary to achieve a desired limit of detection, we computed the probability of species detection from a hypergeometric probability distribution based on a mathematical model of read sampling. The *hypergeometric distribution* is a discrete probability distribution that describes the probability of *k* successes in *n* draws, *without replacement*, from a finite population of size *N* that contains exactly *K* successes, wherein each draw is either a success or a failure. The minimum required read sampling rate can be derived from the distribution as follows.

For a fixed total read count *N* (e.g., 300 million), the probability *P* that species *S* with frequency *f(S)* in the full sample has at least *r* reads in a subsample of *n* (reads) is shown below in Eq. (1):

1 $$P \ge 1 - \mathop {\sum }\limits_{k = 0}^{r - 1} \frac{{\left( {\begin{array}{*{20}{c}} K \\ k \end{array}} \right)\left( {\begin{array}{*{20}{c}} {N - K} \\ {n - k} \end{array}} \right)}}{{\left( {\begin{array}{*{20}{c}} N \\ n \end{array}} \right)}}$$

where *K* = *N·f(S)* is the number of reads originating from *S* in the full sample. This equation can also be used to compute *P*′ as the probability of having at most *r* − 1 reads from *S* among the sampled reads: *P*′ ≥ (1 – *P*). A desired *limit of detection L* for calling species *S* present—e.g., *L* = 100 reads (or *L* = 0.1% of the sampled reads) is needed to define a sufficient read sampling depth, along with the desired probability of success in correctly calling *S* present or absent.

### Sequencing data analysis with the FASER pipeline

This section describes the steps of the FASER pipeline, outlined in Fig. 1, in additional detail. Prior to the matrix analysis, Illumina Universal adapters were removed and reads were trimmed using TrimGalore^34^ (v0.5.0) with a minimum read length parameter 50 bp. The resulting reads were filtered using Kraken^35^ (v1.0), with a custom database built from the PhiX genome (NCBI Reference Sequence: NC_001422.1). Removal of PhiX content is suggested as it is a common contaminant in Illumina sequencing data.^36^ Trimmed non-PhiX reads were used in subsequent analysis.The BLAST^14^ (v2.6.0) search criteria applied were 95% identity over 50% of query length, with e-value threshold of 10^−40^. The projection algorithm by MEGAN CE authors was applied to summarize the read counts at species level.^37^ The promiscuity filtering involves performing two post-processing steps before reporting the BLAST hits:

1. Retain only those BLAST hits per read that have the highest bit score.
2. Identify and remove taxa *S* where <10% of BLAST hits are unique to *S*, i.e., >90% of the hits to *S* also hit other taxa.

Taxonomic labeling from the NCBI RefSeq catalog^15^ was added to the BLAST hits prior to the filtering, with a custom script. After BLAST alignment and custom filtering of promiscuous hits, the remaining hits were filtered to remove non-concordantly paired hits. A hit was determined to be concordant if the left and right paired-end read align against the same database reference sequence, and if the distance between the alignments was at most 1 Mbp allowing concordant reads spanning intron junctions. Further details describing the memory-efficient algorithm for determining concordant pairs are included in Supplementary Methods.

### Constructing simulated datasets

Two simulated food mixtures were created for simulation and testing (Supplementary Table 6). Simulated food mixture 1 comprised of nine eukaryotic animal species with the following number of reads randomly sampled from the respective genomes: 200,000 beef (*Bos taurus*), 200,000 salmon (*Salmo salar*), 100,000 goat (*Capra hircus*), 100,000 lamb (*Ovis aries*), 100,000 black rockcod (*Notothenia coriiceps*), 100,000 chicken (*Gallus gallus*), 1000 duck (*Anas platyrhynchos*), 100 horse (*Equus caballus*), 100 rat (*Rattus norvegicus*), totaling 801,200 matrix synthetic reads. Simulated food mixture 2 contained a mix of randomly sampled plant, animal, and insect synthetic reads that totaled 14.21 M sequence reads: 5 M soybean (*Glycine max*), 4 M rice (*Oryza sativa*), 3 M potato (*Solanum tuberosum*), 2 M corn (*Zea mays*), 200,000 rat (*Rattus norvegicus*), 10,000 drain fly (*Clogmia albipunctata*). In addition, both simulated food mixtures included microbial sequence synthetic reads generated from each of 15 different microbial species: 100 reads per microbe for simulated food mixture 1 and 1000 reads per microbe for simulated food mixture 2. Genomic read simulations were done using DWGSIM^38^ (v0.1.11) with simulated sequencing errors using the following parameters: read length (*l*) = 150, base error rate (*e*) = 0.005, outer distance between the two ends of a read pair (*d*) = 500, rate of mutations (*r*) = 0.001, fraction of indels (*R*) = 0.15, probability an indel is extended (*X*) = 0.3. The details of the chicken (*G*. *gallus*), bovine (*B*. *taurus*), and pork (*S*. *scrofa*) genomes used in simulations of single ingredient food sources are in Supplementary Table 7. Single genome read simulation was performed using DWGSIM with simulated sequencing errors using the same parameters as listed above except *r*, *R*, and *X* were 0 indicating no mutations were added with respect to the reference genomes in these single ingredient simulations.

### Evaluating accuracy of the results

We evaluated the accuracy of FASER species relative quantification in cases where the underlying composition was known. We used median absolute difference to measure the difference between observed and expected species relative proportions. For each true positive species, absolute difference of observed proportion from known proportion was computed. For each false positive species, difference of observed proportion from zero was computed. Median of the absolute differences for true and false positive species in the sample was then computed and reported. The proportion of species-classified reads matching true positive species was computed as the sum of the proportions of all the true positive species in a sample. In the case of tuna sample discussed in the section ‘Accuracy evaluation on single species sequencing data’, the statistics were computed using the species that was present in the database.

In order to compare the differences between all observed vs. expected values for the different matrices to assess accuracy, we used a (one-sided) Pearson’s chi-square test of independence to compare the two sets of values within each matrix. Expected values were derived from known compositions of the eukaryotic matrix and observed values were estimated from FASER results, as explained above. The *χ*^2^ statistic was estimated according to the formula:

$$\chi ^2 = \sum \left( {{\mathrm{observed}}-{\mathrm{expected}}} \right)^2/{\mathrm{expected}}$$

This *χ*^2^ statistic was compared against a table^39^ of *χ*^2^ values and degrees of freedom (calculated as the product of one less each, the numbers of rows and columns) to assess similarity between observed and expected values. Statistically significant differences were assigned at *α* = 0.05.

### Read alignment to chicken, cattle, pig reference genomes

Read alignment to specific food matrix genomes was accomplished using Bowtie 2^40^ (v2.3.4.2) against chicken, cattle, and pig genomes individually (without masking low complexity regions) using sub-sampled 1 million reads per dataset (after trimming and PhiX removal as previously described). Bowtie 2 was configured with default parameters in *very-sensitive-local* mode and considering the primary alignment only. The “proper pair” SAM flag was used to filter the resulting alignments. The number of concordant paired reads aligning to only one genome (exclusive alignments) were determined by a custom command line script.

### Reporting summary

Further information on research design is available in the Nature Research Reporting Summary linked to this article.

## Supplementary information

Supplementary Information

Supplementary Data.

reporting summary.

## Data Availability

All of the sequences generated in this study are available via the 100 K Pathogen Genome Project BioProject (PRJNA186441) (see Supplementary Table 4 for a complete list of accession numbers for each sample).

## Supplementary information

**Supplementary information** accompanies the paper on the *npj Science of Food* website (10.1038/s41538-019-0056-6).

## Code availability

The FASER pipeline and food matrix database were constructed from publicly available tools and reference sequences as described in the Methods section. Automated usability of this pipeline is available through membership in the Consortium for Sequencing the Food Supply Chain.
