# Supplementary material for: Food authentication from shotgun sequencing reads with an application on high protein powders
Source: NPJ Sci Food. 2019 Nov 19;3:24. doi: 10.1038/s41538-019-0056-6 (PMC6863864; doi:10.1038/s41538-019-0056-6)
Supplement: Supplementary file 1 — Supplementary Information [file 41538_2019_56_MOESM1_ESM.pdf]

Supplementary Information for "Food authentication from shotgun sequencing reads with an application on high protein powders**"**
Haiminen *et al.*, npj Science of Food, 2019

Supplementary Methods

Concordant hits filtering for paired reads

The concordancy determination algorithm was designed to be as memory-optimal as possible, and it takes advantage of the fact that BLAST outputs read hits in the same order as the reads in the input file. It takes as input the hits reported for the left input file and the right input file (separate files for the first and second read in the pair) and starts off by reading *B* first hits from the right file into a buffer (ensuring that the last hit in the buffer is also the last hit reported for a read). Next, it reads all hits for the first read in the left input file. If the read is not found in the buffer from the right file, the hits for the read are marked as "possibly concordant" and reported as such, but still removed from the output. If the read is found in the buffer, hits for the read in the left and right files are compared and concordant ones are written to the output. Because of the guaranteed ordering of the read hits, any hits for reads occurring before the concordant matchup can be safely removed from the buffer, and the buffer is next filled up again to *B* hits. The algorithm then proceeds to reading hits for the second read in the left file, looks for concordant hits, and so forth. The algorithm stops when end of file has been reached in the left input file.

Due to memory constraints, it would be infeasible (even on powerful servers) to sort potentially huge input BLAST hits files based on read names or to build lookup tables on read name positions; this would have simplified the concordant filtering algorithm and removed the need to fix a buffer size. We kept track of any reads for which hits for its pair were not found within our selected buffer size of *B* = 50K hits. There were no such cases on the data sets analyzed in this paper, thus in practice we found the buffer size 50K hits sufficient to find the concordant matches.

Timing of the FASER pipeline

In terms of timing of the FASER pipeline, the most time-consuming step is by far the BLAST alignment. On the largest experimental sample that also had a complex matrix including chicken, beef, and pork (MFMB-04), the BLAST alignment took approximately 5.5 hours (median time of alignment per batch when the data was split into a total of six batches). In contrast, the other steps including quality filtering, PhiX filtering, promiscuous hit filtering, concordant taxonomic assignment and relative quantification took approximately 30 minutes in total. The computations were performed on nodes with 24 cores of Intel® Xeon® CPU @ 2.40GHz and 200GB memory. A faster turnaround time could be achieved by further parallelization of the alignment step or by limiting the number of hits that are included in the output.

Supplementary Tables

Sample type	Sequencing type	Read length	Species	
Chicken embryo	RNA (polyA selected)	150 x 2	*Gallus gallus*	https://trace.ncbi.nlm.nih.gov/Traces/sra/?run=SRR1804235
Pork ovaries	RNA (polyA selected)	125 x 2	*Sus scrofa*	https://trace.ncbi.nlm.nih.gov/Traces/sra/?run=SRR6236882
Tuna muscle	RNA (polyA selected)	100 x 2	*Thunnus albacares*	https://trace.ncbi.nlm.nih.gov/Traces/sra/?run=SRR4436659
Carp spleen	RNA (polyA selected)	125 x 2	*Cyprinus carpio*	https://trace.ncbi.nlm.nih.gov/Traces/sra/?run=SRR3239506
Rice root	RNA (polyA selected)	125 x 2	*Oryza sativa*	https://trace.ncbi.nlm.nih.gov/Traces/sra/?run=SRR7079262
Maize leaf	RNA (polyA selected)	100 x 2	*Zea mays*	https://trace.ncbi.nlm.nih.gov/Traces/sra/?run=ERR712359
Poultry meal	Total RNA (MFMB-03)	100 x 2	*Gallus gallus*	https://trace.ncbi.nlm.nih.gov/Traces/sra/?run=SRR7234261
	Shotgun DNA (MFMB-08)	100 x 2		https://trace.ncbi.nlm.nih.gov/Traces/sra/?run=SRR7234262
Meat & bone meal	Total RNA (MFMB-02)	100 x 2	*Bos taurus*	https://trace.ncbi.nlm.nih.gov/Traces/sra/?run=SRR8477337
	Shotgun DNA (MFMB-06)	100 x 2		https://trace.ncbi.nlm.nih.gov/Traces/sra/?run=SRR8477338

Supplementary Table 1: Details for data in Table 3.

Taxon Name	Common name	TaxId	FASER hits	FASER %
Ovis aries	Sheep	89462	115,178	99.58%
Capra hircus	Goat	9925	364	0.32%
Pantholops hodgsonii	Tibetan antelope	9940	109	0.09%
Bubalus bubalis	Water buffalo	59538	8	0.01%
**Total**			115,659	

Supplementary Table 2: Simulated 150K sheep reads FASER pipeline results. Reference sheep genome used: GCF_000298735.2.

Family name	Species scientific name	Species common name	Expected proportion (% weight)	All Food Seq Family hits	FASER Family hits	FASER Species hits	FASER Species Observed %
Anacardiaceae	*Anacardium occidentale*	cashew nut	0.003	1	0	0	0
	Pistacia vera	pistachio	0.01			0	0
Apiaceae	*Apium graveolens*	celery	0.01	17	3	0	0
Betulaceae	*Corylus avellana*	hazelnut	0.1	3	0	0	0
Brassicaceae	*Brassica alba*	mustard	0.1	449	109	59	0.021
Fabaceae	*Arachis hypogaea*	peanut	0.032	62	92	0	
	Glycine max	soybean	0.032			8	0.003
	Lupinus spec.	lupines	0.316			84	0.030
Juglandaceae	*Juglans regia*	walnut	0.316	9	81	81	0.029
Pedaliaceae	*Sesamum indicum*	sesame	0.003	2	3	3	0.001
Rosaceae	*Prunus dulcis*	almond	0.01	6	2	2	0.001

Supplementary Table 3: Plant species observed in sausage calibration experiment. Data used is from All-Food-Seq (Supplemental Table S3 in Ripp et al., BMC Genomics 2014) contrasted with the FASER pipeline results. FASER counts for *Brassica alpa* are from hits against *Brassica napus*, and counts for *Prunus dulcis* from *Prunus mume* hits.

	Sample	BioSample	SRA	Sample Type	
**Preliminary samples**	MFMB02	SAMN10789612	SRR8477337	Meat and bone meal (RNA)	**Paired RNA and DNA of same input material**
	MFMB06	SAMN10789613	SRR8477338	Meat and bone meal (DNA)	
	MFMB03	SAMN09258319	SRR7234261	Poultry meal (RNA)	**Paired RNA and DNA of same input material**
	MFMB08	SAMN09258321	SRR7234262	Poultry meal (DNA)	
**HPP poultry meal samples**	MFMB04	SAMN09258320	SRR8451826	Poultry meal (RNA)	
	MFMB17	SAMN09258322	SRR8451827	Poultry meal (RNA)	
	MFMB20	SAMN09258323	SRR8451828	Poultry meal (RNA)	
	MFMB22	SAMN09258324	SRR8451829	Poultry meal (RNA)	
	MFMB31	SAMN09258325	SRR8451830	Poultry meal (RNA)	
	MFMB33	SAMN09258326	SRR8451831	Poultry meal (RNA)	
	MFMB34	SAMN09258327	SRR8451832	Poultry meal (RNA)	
	MFMB35	SAMN09258328	SRR8451833	Poultry meal (RNA)	
	MFMB38	SAMN09258329	SRR8451824	Poultry meal (RNA)	
	MFMB39	SAMN09258330	SRR8451825	Poultry meal (RNA)	
	MFMB40	SAMN09258331	SRR8451819	Poultry meal (RNA)	
	MFMB41	SAMN09258332	SRR8451820	Poultry meal (RNA)	
	MFMB42	SAMN09258333	SRR8451821	Poultry meal (RNA)	
	MFMB59	SAMN09258334	SRR8451822	Poultry meal (RNA)	
	MFMB65	SAMN09258335	SRR8451815	Poultry meal (RNA)	
	MFMB66	SAMN09258336	SRR8451816	Poultry meal (RNA)	
	MFMB75	SAMN09258337	SRR8451817	Poultry meal (RNA)	
	MFMB78	SAMN09258338	SRR8451818	Poultry meal (RNA)	
	MFMB82	SAMN09258339	SRR8451813	Poultry meal (RNA)	
	MFMB83	SAMN09258340	SRR8451814	Poultry meal (RNA)	
	MFMB84	SAMN09258341	SRR8451837	Poultry meal (RNA)	
	MFMB88	SAMN09258342	SRR8451836	Poultry meal (RNA)	
	MFMB89	SAMN09258343	SRR8451839	Poultry meal (RNA)	
	MFMB92	SAMN09258344	SRR8451838	Poultry meal (RNA)	
	MFMB93	SAMN09258345	SRR8451841	Poultry meal (RNA)	
	MFMB94	SAMN09258346	SRR8451840	Poultry meal (RNA)	
	MFMB95	SAMN09258347	SRR8451843	Poultry meal (RNA)	
	MFMB96	SAMN09258348	SRR8451842	Poultry meal (RNA)	
	MFMB97	SAMN09258349	SRR8451835	Poultry meal (RNA)	
	MFMB98	SAMN09258350	SRR8451834	Poultry meal (RNA)	
	MFMB99	SAMN09258351	SRR8451823	Poultry meal (RNA)	

Supplementary Table 4: MFMB sample details.

Common name	Scientific name	Source
Barley	*Hordeum vulgare*	ftp://ftp.ncbi.nlm.nih.gov/genomes/all/GCA/000/326/125/GCA_000326125.1_ASM32612v1
Cockroach	*Blattella germanica*	ftp://ftp.ncbi.nlm.nih.gov/genomes/all/GCA/000/762/945/GCA_000762945.1_Bger_1.0
Drain fly	*Clogmia albipunctata*	ftp://ftp.ncbi.nih.gov/genomes/genbank/invertebrate/Clogmia_albipunctata/latest_assembly_versions/GCA_001014945.1_ASM101494v1
Tuna	*Thunnus orientalis*	ftp://ftp.ncbi.nlm.nih.gov/genomes/all/GCA/000/418/415/GCA_000418415.1_Thunnus_orientalis_ver_Ba_1.0
Wheat	*Triticum aestivum*	ftp://ftp.ensemblgenomes.org/pub/release-39/plants/fasta/triticum_aestivum/dna/

Supplementary Table 5: Additional 5 references added to Blast Database. All genomes are version 1 for the species.

	Simulated Food Mixture 1				
	**Common name**	**Genus and species**	**Number of synthetic reads**	**Percentage of matrix**	**Identifier**
1	Domestic beef	*Bos taurus*	200,000	24.963%	GCF_000003205.7_Btau_5.0.1_genomic.fasta.gz
2	Salmon	*Salmo salar*	200,000	24.963%	GCF_000233375.1_ICSASG_v2_genomic.fasta.gz
3	Goat	*Capra hircus*	100,000	12.481%	GCF_001704415.1_ARS1_genomic.fasta.gz
4	Lamb	*Ovis aries*	100,000	12.481%	GCF_000298735.2_Oar_v4.0_genomic.fasta.gz
5	Black rockcod	*Notothenia coriiceps*	100,000	12.481%	GCF_000735185.1_NC01_genomic.fasta.gz
6	Chicken	*Gallus gallus*	100,000	12.481%	GCF_000002315.4_Gallus_gallus-5.0_genomic.fasta
7	Mallard duck	*Anas platyrhynchos*	1,000	0.125%	GCF_000355885.1_BGI_duck_1.0_genomic.fasta.gz
8	Horse	*Equus caballus*	100	0.012%	GCF_000002305.2_EquCab2.0_genomic.fasta.gz
9	Norway rat	*Rattus norvegicus*	100	0.012%	GCF_000001895.5_Rnor_6.0_genomic.fasta.gz
10	Pseudomonas aeruginosa	*Pseudomonas aeruginosa*	100		Pseudomonas_aeruginosa_GCF_000006765.1_ASM676v1_genomic.fna
11	Vibrio cholerae	*Vibrio cholerae*	100		Vibrio_cholerae_000006745.fasta
12	Streptococcus pyogenes	*Streptococcus pyogenes*	100		Streptococcus_pyogenes_000006785.fasta
13	Streptococcus pneumoniae	*Streptococcus pneumoniae*	100		Streptococcus_pneumoniae_000006885.fasta
14	Staphylococcus aureus	*Staphylococcus aureus*	100		Staphylococcus_aureus_000009005.fasta
15	Salmonella enterica	*Salmonella enterica*	100		Salmonella_enterica_000006945.fasta
16	Peptoclostridium difficile	*Peptoclostridium difficile*	100		Peptoclostridium_difficile_000009205.fasta
17	Neisseria meningitidis	*Neisseria meningitidis*	100		Neisseria_meningitidis_000008805.fasta
18	Mycobacterium avium	*Mycobacterium avium*	100		Mycobacterium_avium_000007865.fasta
19	Listeria monocytogenes	*Listeria monocytogenes*	100		Listeria_monocytogenes_000008285.fasta
20	Klebsiella pneumoniae	*Klebsiella pneumoniae*	100		Klebsiella_pneumoniae_000009885.fa
21	Escherichia coli	*Escherichia coli*	100		Escherichia_coli_000005845.fasta
22	Enterococcus faecium	*Enterococcus faecium*	100		Enterococcus_faecium_000174395.fa
23	Campylobacter jejuni	*Campylobacter jejuni*	100		Campylobacter_jejuni_000009085.fa
24	Acinetobacter baumannii	*Acinetobacter baumannii*	100		Acinetobacter_baumannii_000018445.fa
	**Matrix total**		**801,200**		
	**TOTAL**		**802,700**		

Supplementary Table 6a: Simulated Food Mixture 1 composition.

	Simulated Food Mixture 2				
	**Common name**	**Genus and species**	**Number of synthetic reads**	**Percentage of matrix**	**Identifier**
1	Soybean	*Glycine max*	5,000,000	35.186%	GCF_000004515.4_Glycine_max_v2.0_genomic.fasta.gz
2	Japanese rice	*Oryza sativa Japonica Group*	4,000,000	28.149%	GCF_001433935.1_IRGSP-1.0_genomic.fasta.gz
3	Potato	*Solanum tuberosum*	3,000,000	21.112%	GCF_000226075.1_SolTub_3.0_genomic.fasta.gz
4	Corn	*Zea mays*	2,000,000	14.075%	GCF_000005005.2_B73_RefGen_v4_genomic.fasta.gz
5	Norway rat	*Rattus norvegicus*	200,000	1.407%	GCF_000001895.5_Rnor_6.0_genomic.fasta.gz
6	Drainfly	*Clogmia albipunctata*	10,000	0.070%	GCA_001014945.1_ASM101494v1_genomic.fasta.gz
7	Pseudomonas aeruginosa	*Pseudomonas aeruginosa*	1,000		Pseudomonas_aeruginosa_GCF_000006765.1_ASM676v1_genomic.fna
8	Vibrio cholerae	*Vibrio cholerae*	1,000		Vibrio_cholerae_000006745.fasta
9	Streptococcus pyogenes	*Streptococcus pyogenes*	1,000		Streptococcus_pyogenes_000006785.fasta
10	Streptococcus pneumoniae	*Streptococcus pneumoniae*	1,000		Streptococcus_pneumoniae_000006885.fasta
11	Staphylococcus aureus	*Staphylococcus aureus*	1,000		Staphylococcus_aureus_000009005.fasta
12	Salmonella enterica	*Salmonella enterica*	1,000		Salmonella_enterica_000006945.fasta
13	Peptoclostridium difficile	*Peptoclostridium difficile*	1,000		Peptoclostridium_difficile_000009205.fasta
14	Neisseria meningitidis	*Neisseria meningitidis*	1,000		Neisseria_meningitidis_000008805.fasta
15	Mycobacterium avium	*Mycobacterium avium*	1,000		Mycobacterium_avium_000007865.fasta
16	Listeria monocytogenes	*Listeria monocytogenes*	1,000		Listeria_monocytogenes_000008285.fasta
17	Klebsiella pneumoniae	*Klebsiella pneumoniae*	1,000		Klebsiella_pneumoniae_000009885.fa
18	Escherichia coli	*Escherichia coli*	1,000		Escherichia_coli_000005845.fasta
19	Enterococcus faecium	*Enterococcus faecium*	1,000		Enterococcus_faecium_000174395.fa
20	Campylobacter jejuni	*Campylobacter jejuni*	1,000		Campylobacter_jejuni_000009085.fa
21	Acinetobacter baumannii	*Acinetobacter baumannii*	1,000		Acinetobacter_baumannii_000018445.fa
	**Matrix total**		**14,210,000**		
	**TOTAL**		**14,225,000**		

Supplementary Table 6b: Simulated Food Mixture 2 composition.

**Food matrix**	**Scientific name**	**Version**	**Source**
Chicken	*Gallus gallus*	5	ftp://ftp.ncbi.nlm.nih.gov/genomes/all/GCF/000/002/315/GCF_000002315.4_Gallus_gallus-5.0
Beef	*Bos taurus*	5.0.1	ftp://ftp.ncbi.nlm.nih.gov/genomes/all/GCF/000/003/205/GCF_000003205.7_Btau_5.0.1/
Pork	*Sus scrofa*	10.2	ftp://ftp.ncbi.nlm.nih.gov/genomes/all/GCF/000/003/025/GCF_000003025.5_Sscrofa10.2/

Supplementary Table 7: Reference genomes for read alignment.
